# Social Support and Health Services Use in People Aged over 65 Years Migrating within China: A Cross-Sectional Study

**DOI:** 10.3390/ijerph17134651

**Published:** 2020-06-28

**Authors:** Chengxu Long, Ruoxi Wang, Da Feng, Lu Ji, Zhanchun Feng, Shangfeng Tang

**Affiliations:** 1 School of Medicine and Health Management, Tongji Medical College, Huazhong University of Science and Technology, 13 Hangkong Road, Wuhan 430030, Hubei, China; longchengxu@hust.edu.cn (C.L.); ruoxiwang@hust.edu.cn (R.W.); jilu2018@hust.edu.cn (L.J.); 2 School of Pharmacy, Tongji Medical College, Huazhong University of Science and Technology, 13 Hangkong Road, Wuhan 430030, Hubei, China; fengda@hust.edu.cn

**Keywords:** essential public health services, health behavior, health services utilization, elderly migrants, China

## Abstract

Background: Due to the household registration system, Chinese elderly migrants have insufficient access to health services and social support. Thus, this study examined the use of health services, the access to social support, and the interaction among the elderly migrating within China. Methods: Data were obtained from the China Migrant Dynamic Monitoring Survey in 2015, adopting probability proportionate to size as the sampling strategy. Structural equation modeling and mediating effect tests were employed to explore the associations. Results: Approximately 45.9% of elderly migrants did not seek health services when needed. The use of outpatient and inpatient services was more common than free essential public health services. The use of health services was negatively associated with migrating duration and migrating for offspring, while it was positively associated with outer social support. The mediating effects of outer social support were discovered on the relationships between the use of health services and independent variables such as migrating duration and migrating for offspring, respectively. Conclusion: Elderly migrants with a longer migrating duration or migrated for offspring seem to obtain less outer social support, resulting in a decreased use of health services. Outer social support was suggested as a key effort to improve the equalization of health services in Chinese elderly migrants.

## 1. Introduction

Since the new Chinese health system reform was launched in 2009, the government has increased the budget in primary healthcare delivery. One of the vital policies was the equalization of Essential Public Health Service (EPHS), which aims to ensure that Chinese citizens have equal access to essential services and to satisfy primary health needs. The package of EPHS, which mainly includes annual physical examinations and free follow-up services for those diagnosed with hypertension or diabetes, is free for people aged 65 or above since 2009 [1]. In China, while management and guidance are provided by the administrative health departments and higher-level organizations such as the Centers for Disease Control and Prevention and hospitals for chronic diseases [2], the essential health services are mainly provided by community healthcare centers with subordinate clinical stations in urban areas and by township hospitals with subordinate village clinics in rural areas [3].

The Chinese elderly migrants refer to the elderly who migrated for caring for the younger generations, employment, and other causes within China, such as the acceleration of urbanization [4]. The reports showed that the population of the Chinese elderly migrants increased from 5.03 million to 13.04 million with an annual increase of 6.6% from 2000 to 2015 [5]. China is a country with rapid economic development, and the migrating population also has its specificity in historical characteristics. The Hukou system is the household registration system in China to provide a foundation for the population administrative policy and public services. It links people’s healthcare and public welfare to their household registration area. In fact, the majority of migrants have health insurance in their previous area without local health insurance [4,6]. In addition to a lack of proactive behavior in seeking health services, elderly migrants also suffer from limited access to healthcare information [7]. Hence, the Hukou system may result in an exclusion against elderly migrants, since most Chinese elderly migrants migrated for supporting offspring rather than receiving support from their family. The social support in this study is measured by medical insurance and local friends, indicating social support besides family support. Besides the lack of local medical insurance, the elderly migrants have to reconstruct their social networks. Thus, the issue of social support and the use of health services for Chinese elderly migrants are more crucial than ever.

To deal with the increasing elderly migrant population, the “Active Aging—Policy Framework” recommended three directions, such as health, participation, and preservation [8,9]. The Chinese government’s migration policy adjustment has gone through three stages: a liberalization of entry into the city, an addressing of equal rights, and a comprehensive promotion of urbanization [5,10]. Several studies focused on the associations among the migrating factors, medical insurance, and the use of health services for working-aged migrants [4,11,12]. Nevertheless, within the context of population migration, the issue of the intergenerational connection between outer social support and the use of health services among elderly migrants is under-investigated. Therefore, it still needs further study to develop a model to identify the associated factors and the pathway of the health services use among Chinese elderly migrants. As such, this study aims to establish a health services use model for the Chinese elderly migrants through structural equation modeling (SEM) and estimate the interactions among migration, outer social support, and health services use.

## 2. Theoretical Backgrounds and Hypothesis Development

### 2.1. Health Service Use

Health services use is an objective indicator to describe the comprehensive description of a health service system, and reveal the interaction between the demand side and the supply side of healthcare within disease prevention, medical treatment and rehabilitation [11]. It directly describes the number of health services provided by the supply side and the population’s health demand. Simultaneously, it also indirectly determines the impact of health services delivery systems on the residents’ physical health status. The health service utilization indicators include the use of outpatient services (OS), inpatient service, and EPHS [4,12].

### 2.2. Population Migration

Population migration refers to the change in population distribution in two characteristics: time and space [12]. Population migration is a part of the government’s strategy to promote industrialization, urbanization, and poverty alleviation, thereby rebuilding the family structure, distribution, and living arrangement. Duration is one of the core dimensions of migration. Migrating time attributes generally refers to the short or long-term migration into the immigration area [13]. Population migration hits the previous social network and health behaviors, and the registering systems restrict elderly migrants from seeking health services [14]. The longer the migrating duration, the more challenges there will be for the health services, such as in both medical treatment and off-site medical insurance settlement [15]. Thus, this study proposes the hypothesis:

**Hypothesis 1** **(H1).** *Migrating duration is negatively associated with the use of health services in elderly migrants in China.*

### 2.3. Social Support

**Figure 1:**
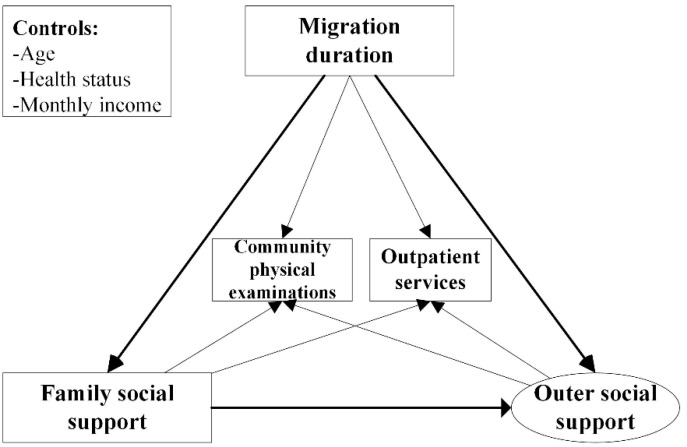
Conceptual model.

The social convey model provides the implications on how to construct a theoretical framework for explaining the interactions among migration, social support, and health services use [9,16], more details presented in Figure 1. According to the social convey model, the social support of the elderly can be expressed in concentric circles [9]. The inner-circle refers to the closest individuals related to the elderly, while the outer circle denotes the less close social partners. Given that in the context of the unique Chinese culture, Chinese elderly migrants are more likely to provide more support to their offspring rather than receive support. Thus, this study focused on exploring the interactions of outer social support since family support is not applicable to most of the Chinese elderly migrants, comparatively. Besides, with the unique characteristics of guardians, seniors tend to live or even migrate with the family, enjoying the role of guardians in the family. Migrating for offspring refers to some seniors for whom the main reason for migration was for caring for the younger generations, indicating the intergenerational relationship [4]. Evidence showed that the seniors that migrated for offspring have more intergenerational interactions than those separated from their children, giving more advice to their children and also receiving the support and comfort from the family in return [17,18]. Thus, we propose the hypothesis:

**Hypothesis 2** **(H2).** *Migrating for offspring is positively associated with the use of health services in elderly migrants in China.*

In the outer cycle, this study explored medical insurance and local friends, indicating formal and informal social support. Formal support was led by the government or formal non-governmental organizations. Health insurance is a representative indicator of formal social support concerning personal healthcare security. Lack of health insurance in the immigration area elites a direct negative effect with the insufficient use of EPHS (essential public health services). On the other hand, informal support was defined in terms of personal networks or communities, including the breadth of the individual’s social network and the degree to which the network is supported. Evidence showed that the number of local friends represented the informal social support and resources among elderly migrants [17,19]. According to the diffusion theory, social learning and social influence come into being when the interaction is embedded in social networks. Thus, health services information could be obtained through observation and interpersonal communication within one’s social network. This means that elderly migrants are more likely to seek advice from peers and friends, resulting in the shaping of their health behaviors when the extant experience cannot solve their problems [20]. Meanwhile, with respect to the impact of group members on personal preferences and decisions, social influence also has an impact on individual decisions in the form of social pressure. As demonstrated in previous studies, the influence of peers and friends increases the likelihood to choose health services [21,22]. As such, we proposed the following research hypotheses:

**Hypothesis 3** **(H3).** *Outer social support is positively associated with the use of health services in elderly migrants in China.*

**Hypothesis 4** **(H4).** *Outer social support has a mediating effect on the relationship between migrating duration and the use of health services.*

**Hypothesis 5** **(H5).** *Outer social support has a mediating effect on the relationship between migrating for offspring and the use of health services.*

## 3. Methods

### 3.1. Data Sampling

This study included migrants aged over 65 years for the SEM analysis (N = 11,161). Since hypertension and diabetes are the representative chronic diseases in China [23] and they are included in the EHPS package [24], this study selected those elderly individuals who needed inpatient services (N = 1169) and those with hypertension/diabetes (N = 2606) for descriptive analysis. As Table 1 illustrates, the mean age of the total 11,161 participants was 71.18 ± 6.11 years, and the mean migrating duration was 10.66 ± 6.40 years. The majority of participants had a lower educational experience, with a monthly household income of less than CNY 2000, not migrated for offspring, and with access to health insurance and local friends.

The national annual cross-sectional dataset was openly applied from the Migrant Population Service Center, National Health Commission that conducted the Chinese Migrant Dynamic Monitoring Survey in 2015. Based on the annual report of migrants from 32 provinces/autonomous districts/direct-controlled municipalities, the stratified multi-stage scale-oriented probability proportionate to size method was employed to collect the data. To investigate the outer social support and the use of health services in elderly migrants, this study selected the individuals aged 65 or above due to the annual physical examination services, which only covered the people aged 65 or above for free. As reported in the data specification, the participants filled in the questionnaires anonymously, and the quality of the data was guaranteed through the reasonable sampling process, without any deletion. Specific ethics approval was not required for this study since it was a secondary analysis of a publicly available dataset. Details are available on the website: http://www.chinaldrk.org.cn/wjw/#/application/index.

### 3.2. Measurements

The constructs and the items with assignments are presented in Table 2. To ensure the validity of this study, the measurement items were adapted from previous studies as follows. The items measuring the use of community physical examinations (CPE) were taken from Chen et al. [1]. The items measuring migrating duration, migrating for offspring, and the use of outpatient service (OS) were adapted from Zhang et al. [4]. Items measuring outer social support were adapted from Li et al. and Seid et al. [9,10]. In this investigation, the use of OS was measured by the rate of visiting physicians, and the use of inpatient services was measured by the hospitalization rate. Migrating duration refers to the time of the migrants’ short or long-term migration across administrative boundaries in space. Migrating for offspring refers to the main reason for which the seniors migrated was because of caring for the younger generation, indicating the intergenerational family relationship. The use of EPHS in elderly migrants was measured by the physical examination rate of the population and the utilization of follow-up services among elderly migrants with hypertension or diabetes. Physical health status, monthly household income, and sex were set as the control variables.

### 3.3. Analytical Methods

The analytical strategy combined descriptive statistics, the chi-square test, structural equation modeling (SEM), and mediating effects analysis. Chi-square tests were employed to describe the frequency, percentage, and difference among the elderly migrants with different age groups. The SEM with Amos 21.0 software (IBM, Almond, the U.S.A) was used to construct the models, and to estimate the associations of the latent variables, such as outer social support, on the use of health services in elderly migrants. The maximum likelihood method was employed in the parameter estimation. The following criteria were adopted to evaluate the hypothetical possible path and model fitness: the value of χ^2^/df less than 3 indicates that the hypothetic model has good fit indexes. Although the χ^2^/df value in this study is slightly greater than 3, it can still be empirically regarded as a well fitted structural model due to its large sample size [25,26]. A goodness-of-fit ndex (GFI) and an adjusted goodness-of-fit index (AGFI) over 0.90 indicates a well fitted model for the data [27]. The value of the root mean square error of approximation (RMSEA) and root mean square residual (RMR) less than 0.05 show a good model fit. The critical ratio (C.R.) = intercept/(unstandardized path coefficient/its standard error) was calculated. A difference with an absolute value of C.R. over 1.96 and a value of *p* less than 0.05 were considered significant.

Mediating effect tests were applied to explore the interactions between the use of health services and the different levels of observable variables. There are several conditions for the mediating effects to be satisfied: the link between the independent variable and the dependent variable is significant; the link between the independent variable and the mediator is significant; the link between the mediator in the regression model that contains the independent variable and the mediator is significant [25].

## 4. Results

### 4.1. The Use of Health Services

Approximately 66.2% of elderly migrants did not receive the CPE in the past year of investigation. Only one-third of those with hypertension/diabetes were followed up, which required four exams per year in accordance with the national manual. In terms of the health services payment, approximately 45.9% of the respondents did not visit physicians when they suffered from illness. Details are shown in Table 3. Among those with disease/injury and that should be hospitalized, 18.0% did not receive inpatient services in the past year. Among those who did not use inpatient services, 38.1% attributed the lack of use to the fact that their family members thought it was unnecessary, while 3.3% attributed the lack of use to the fact that their family members failed to care if they were hospitalized.

### 4.2. Test of Models

Based on the empirical research in SEM, at least one path should be referred and its coefficient should be set as 1 when the latent variable is measured by multiple observable variables [27]. Thus, to estimate the effect of outer social support on the use of CPE, the coefficient of the path from outer social support to OS was set as 1. Conversely, the coefficient of the path from outer social support to CPE was set as 1 to estimate the effect of outer social support on the use of OS. The fit indices of both models were the same and are shown in Table 4. In comparison to the previously recommended values [25,27], the χ^2^/df (5.913) in this study was slightly greater than 3 due to the large sample size’s (N = 11,161) sensitivity, whereas the other indices indicated that the model was acceptable with the comparison of the empirical data.

**Figure 2:**
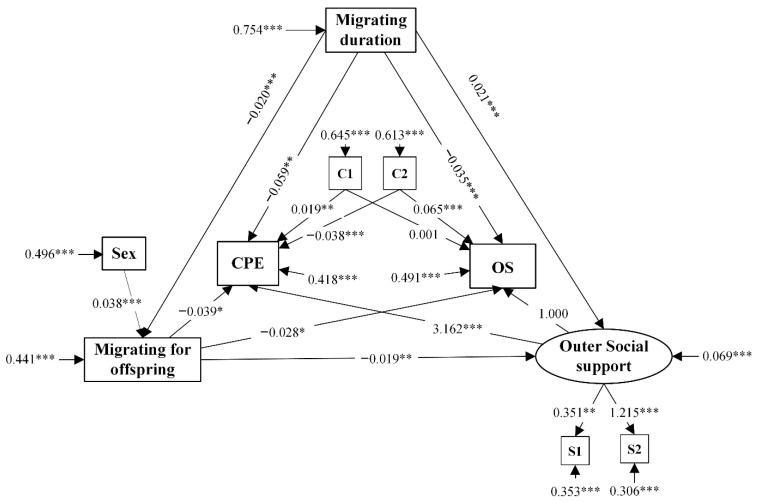
Structural model 1 towards the CPE. CPE, community physical examinations; OS, outpatient services; C1, health status; C2: monthly household income; S1, health insurance; S2, local friends. *, *p* < 0.05; **, *p* < 0.01; ***, *p* < 0.001.

In order to test the aforementioned hypothesis regarding the use of CPE, the standardized coefficient of the specified paths, as well as the control variables and their standard errors, were captured in Model 1 and reported in Figure 2. To test the validity of the measurement, the significantly standardized loadings of this model were reported in Table 5. The observed variables such as health insurance and local friends were loaded on the latent variables, such as outer social support, and the loading was over 0.3 which indicated that the selected variables effectively represented the latent variable.

Migrating duration demonstrated a directly negative effect on the use of CPE, while outer social support demonstrated a directly positive effect (H1 and H3). However, the migrating for offspring showed a directly negative effect on outer social support, which was in stark contrast to the H2. The coefficients of the path from migrating duration and migrating for offspring to the use of CPE were observed in the study, and the mediating effects of outer social support were also reported (H4 and H5). In other words, elderly migrants with longer migrating duration or that migrated for offspring seem to receive less outer social support, resulting in a decreased CPE utilization. The control variables, such as health status, positively influenced the use of CPE and the use of OS, while household income demonstrated a negative effect on the use of CPE. Sex demonstrated a positive effect on migrating for offspring.

**Figure 3:**
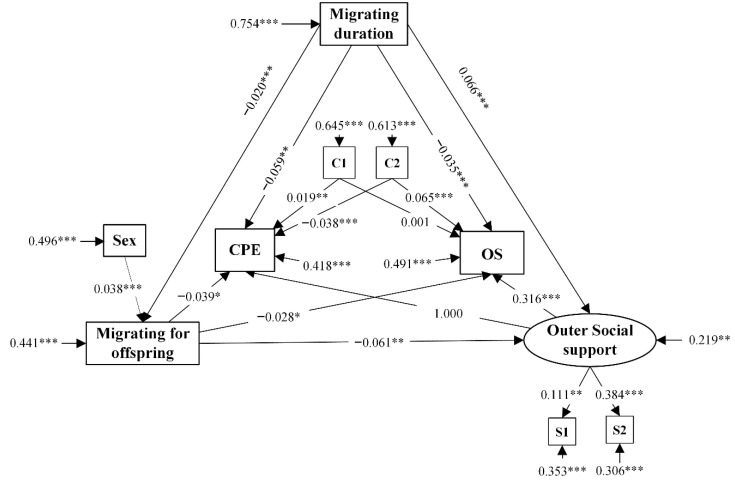
Structural model 2 towards OS. CPE, community physical examinations; OS, outpatient services; C1, health status; C2: monthly household income; S1, health insurance; S2, local friends *, *p* < 0.05, **, *p* < 0.01, ***, *p* < 0.001.

In terms of testing the aforementioned hypothesis regarding the use of OS, the standardized estimates for the specified paths, as well as the control variables and standard errors, were reported in Figure 3. A similar effect of the paths from the migrating duration, migrating for offspring, and outer social support to the use of OS was also captured in Table 6. Interestingly, the coefficient of the path from outer social support to the use of CPE (B = 3.162) was much greater than that from the outer social support to the use of OS (B = 0.316).

### 4.3. Test of Mediating Effects

The mediating effects analysis was employed to test the effect of outer social support on the use of health services. As shown in Table 7, all of the mediating effects of outer social support (measured by health insurance and local friends) were significant, from migrating for offspring and migrating duration to the use of health services. Thus, the first and second conditions for the mediating effect were satisfied. Both relations from migrating duration to the mediators (local friend or health insurance) were significant, and the link between the use of outpatient services and the migrating duration is smaller than that of the mediators (local friend or health insurance) and migrating duration. As such, both local friends and health insurance partially mediated the effect of migrating duration on the use of outpatient services. Similarly, local friends partially mediated the effect of migrating for offspring on the use of community physical examinations. Simultaneously, this study also tested the association among migrating for offspring, sex, and health services use. The mediating effect of sex between migrating for offspring and health services use was not discovered.

Finally, the results of hypotheses testing are summarized in Table 8. H1, H3, H4, and H5 were supported, while H2 was unsupported in this study.

## 5. Discussion

This study investigated differences in the use of different health services by elderly migrants within China, and estimated the effects of the associations and their interaction pathway, concluding with the findings as follows: the use of essential public health services was insufficient compared with the use of outpatient or inpatient services in elderly migrants; migrating duration and migrating for offspring were negatively associated with the use of community physical examinations, while outer social support demonstrated a directly positive effect; and the mediating effect of outer social support was observed in the relations between the use of health services and independent variables including migrating duration and migrating for offspring.

This study found that those that migrated for offspring received insufficient outer social support and restrained the use of health services, whereas outer social support partially mediated this negative association. The insufficient use of public health services might be related to weak awareness and ineffective equalization in preventive care. Currently in China, although the health-centered health delivery system has been widely endorsed in recent years, the prevention-oriented model has not actually been fully established. Public health services were mainly provided by the community health service centers, while the migrants prefer to seek health services from subordinate clinical stations due to increased accessibility with regards to economic and space concerns. Moreover, with the unique characteristics of guardians, Chinese parents tend to be more concerned about their descendants and enjoy the role of caregiver in the family. Thus, they are more likely to provide more support to their offspring rather than receive support [9]. With the beliefs of selfless dedication, elderly migrants that migrated for offspring may ignore or sacrifice their health needs so as to care better for their younger generations [28]. Additionally, the intergenerational conflicts may also undermine the well being and the use of health services among the senior population [29,30]. Those with better intergenerational relationships are more likely to receive care at home [31,32]. The female is more likely to migrate for caring for younger generations. Whereas, the sex did not mediate the relationship between migrating for offspring and the use of health service. The main factors related to health services utilization among those that migrated for offspring would be the demand, income, etc., instead of sex [4,33].

Interestingly, this study also discovered a significant influence of outer social support on the use of public health services in comparison with outpatient services utilization. It might be explained by the exclusive features of the services and the elasticity demand on the services. The public health services are available and free to everyone, while outpatient services are in rigid demand with small elasticity. Thus, the sensibility varies from public services to medical services, and the price of the services might exclude the groups who are poor or with a lower willingness to pay. The health services with a price were also popular to use in the context of reimbursement, which was consistent with previous studies concluding that the elderly with a poor socioeconomic status are more likely to use OS with local insurance and family support [34,35]. The evidence also demonstrates that outer social support contributes to releasing the suppressed demand, or in other words, the seniors covered by insurance are encouraged to utilize more health services than those without health insurance [36].

Simultaneously, we found that health insurance partially mediated the negative relationship between the use of outpatient services and migrating duration. It might be explained by the reimbursement disparity among the health insurance policies [37] since the out-of-pocket nature of healthcare and health insurance reimbursement are the vital issues for their decision on visiting physicians [38,39]. Evidence shows that merely 85.2% of the elderly migrants had health insurance and 88.5% of migrants had to receive health insurance from their household registration [23]. Compared with the migrants, evidence shows that outer social support also plays a mediating role in OS utilization since local residents with local health insurance are more likely to have larger social networks [4]. In the context of Chinese culture, the elderly regard living with offspring as a burden to their family due to the hectic routine of their offspring. This elderly population migrating for offspring shows a sign that the elderly have the initiative to migrate and their families are more likely to have a comparatively better socio-economic characteristic to invite the elderly to the immigration area. It would be affordable for these elderly migrants to receive OS, while health insurance is inclined to support primary health services. Hence, without affordable and reasonable health insurance policies targeting migrants, the healthcare equalization objective is still difficult to achieve even with an incremental number of health services [40].

However, the effect of outer social support on the use of public health services should not be ignored, given that those with more outer social support were more likely to use the services. The elderly’s health behavior is largely dependent on their subjective consciousness despite their health need. The use of public health services is easily influenced by the surrounding environment [4]. Local friends, as a trusted information source [41], play an important role in the transmission of an individual’s health behaviors and knowledge [42].

As we hypothesized, local friends partially mediated the relationship not only between the use of outpatient services and migrating duration but also between the use of community physical examinations and migrating for offspring. The concept of assimilation could explain the behavior of migrants seeking health services [34,43]. Elderly migrants have limited access to health services due to the Hukou system. Meanwhile, with the complexity of the living environment and conservative consciousness dismissed to adopt new circumstances and health behaviors, the dilemma of loneliness and the difficulty of finding a companion in a new environment were quite common among elderly migrants reconstructing social networks [4]. With the migrating duration extended, migrants are gradually integrated into the society in the immigration areas, new ideas are adopted selectively, and health behaviors may change accordingly [42]. More specifically, the social convey model suggests that the closer to the inner of the social network circle, the higher the intimacy. The degree of emotional intimacy depends on the number of friends which plays a role in complementing and supporting the inner intimacy [28]. Seniors prefer to seek comfort from friends rather than offspring, to address family stress and alleviate the pressure of intergenerational conflicts, which might be the internal mechanism to improve health services utilization.

As such, the delivery capacity of preventive care at the grassroots level and equalization efforts of health services for elderly migrants should be improved. Outer social support is suggested as a key effort to improve the use of health services among elderly migrants. Family members should enhance positive intergenerational communication and construct a harmonious intergenerational relationship. Seniors are encouraged to discuss their health needs and to not regard seeking health services as a burden to the family [9]. At the community level, committees and friends should help provide the instrumental and emotional support to the elderly migrants in terms of health services utilization. On-site consultation is suggested to help obtain health information and develop healthy behaviors. The government should further implement the Hukou reform, conduct social work intervention for migrants, advance the equalization of health services, and advocate a traditional culture of filial piety in the society.

To the best of our knowledge, this study focused on Chinese elderly migrants, investigated the interaction pathway of outer social support in the use of health services, and explored the interactions between the use of health services, migrating for offspring, and outer social support in the context of population migration. This study contributed to the development of new models that identify the factors related to the use of health services among Chinese elderly migrants and provided suggestions for improving its utilization and social work for the elderly migrants.

## 6. Limitation

This study has several limitations that should be considered. Since the last survey on elderly migrants was in 2015, our findings reflect the situation in that year. The cross-sectional data cannot indicate trends or long-term associations between the use of health services and relevant indicators. These observational data may be subject to unmeasured confounders, and therefore cannot be used to draw causal inferences. It needs further research to explore the potential impact of more controlled variables. Due to the limit of the public database, it still needs further research to explore the comprehensive social support involved in family support and community support and to employ different ways of measurements instead of binary variables.

## 7. Conclusions

In this study, we estimated the differences in health services utilization among elderly migrants, emphasized exploring the interaction between outer social support and the use of health services in the context of population migration, and therefore, proposed references for improving the use of health services and social work for elderly migrants. As the findings of this study concluded, the use of public health services was insufficient in comparison to outpatient or inpatient services among elderly migrants. The mediating effect of outer social support was observed among migrating duration, migrating for offspring, and health services use. Elderly migrants with a longer migrating duration or that migrated for offspring seem to obtain less outer social support, resulting in a decreased use of health services. Outer social support is suggested as a key effort to improve the equalization of health services in Chinese elderly migrants.

## Figures and Tables

**Table 1:** Basic characteristics of the participants (n, %).

Variable	Item	N = 11,611	%
Age (years)	Mean (SD)	71.18 ± 6.11	
Sex	Male	6228	55.8
	Female	4933	44.2
Marital status	Single	1547	13.9
	Currently married	9614	86.1
Education	Primary school and below	4223	37.8
	Middle school	3902	35.0
	High school and above	3036	27.2
Health status	Healthy	4821	43.2
	Generally healthy	5019	45.0
	Unhealthy	1321	11.8
Monthly household income	<CNY 2000	5321	47.7
	CNY 2000–5000	4725	42.3
	>CNY 5000	1115	10.0
Migrating duration (years)	Mean (SD)	10.66 ± 6.40	
	<5	2354	21.1
	5–10	4413	39.5
	>10	4394	39.4
Migrating for offspring	Yes	3756	66.3
	No	7405	33.7
Health insurance	Yes	9510	85.2
	No	1651	14.8
Local friends	Yes	9856	88.3
	No	1305	11.7

**Table 2:** Construct measurement.

Construct	Item	Measurement	Source
Use of CPE	CPE: Whether to receive community annually free physical examination?	1 = no; 2 = yes	[1]
Use of OS	OS: What will you prefer to do when you suffered from common illnesses?	1 = The other choices; 2 = Visit physicians	[4]
Migrating duration	The duration for this migration. (years)	1 ≤ 5; 2 = 5–10; 3 ≥ 10	[4]
Migrating for offspring	The reason for this migration.	1 = The other choices, 2 = Caring for offspring	[9,19]
Outer social support	S1: Do you have medical insurance?	1 = no; 2 = yes	[9,10]
	S2: Do you have local friends?	1 = no; 2 = yes	
Control variable	C1: Physical health status	1 = healthy; 2 = general healthy; 3 = unhealthy	
	C2: Monthly household income after tax	1 ≤ CNY 2000; 2 = CNY 2000–5000; 3 ≥ CNY 5000	

OS, outpatient services; CPE, community physical examinations.

**Table 3:** Health services use in elderly migrants, n (%).

	Health Services use	Yes	No
Free	CPE	3770(33.8%)	7391(66.2%)
	Hypertension/diabetes follow-up	913(35.0%)	1693(65.0%)
Unfree	OS	5120(45.9%)	6041(54.1%)
	Inpatient services	959(82.0%)	210(18.0%)

OS, outpatient services; CPE, community physical examinations.

**Table 4:** Summary of the fit indices.

Fit Indices	χ^2^/df	GFI	AGFI	RMSEA	RMR
Recommended value	<3	>0.90	>0.80	<0.05	<0.05
Value in the models	5.913	0.976	0.938	0.043	0.002

df, degree of freedom; GFI, goodness-of-fit index; AGFI, adjusted goodness-of-fit index; RMSEA, root mean square error of approximation; RMR, root mean square residual.

**Table 5:** Path coefficients for the hypothetic model 1 towards CPE.

Path Constructs		Coefficient	S.E.	C.R.	*p*
Independent Variable	Dependent Variable				
migrating duration	migrating for offspring	−0.020	0.006	−3.566	<0.001
migrating duration	outer social support	0.021	0.005	4.532	<0.001
migrating for offspring	outer social support	−0.019	0.007	−2.953	0.003
outer social support	health insurance	0.351	0.107	3.282	0.001
outer social support	local friends	1.215	0.190	6.393	<0.001
outer social support	CPE	3.162	0.818	3.864	<0.001
migrating duration	CPE	−0.059	0.020	−2.982	0.003
migrating duration	OS	−0.035	0.008	−4.567	<0.001
migrating for offspring	CPE	−0.039	0.025	−1.595	0.036
migrating for offspring	OS	−0.028	0.013	−2.167	0.030
health status	CPE	0.019	0.007	2.723	0.006
health status	OS	0.001	0.008	0.095	0.924
household income	CPE	−0.038	0.008	−4.842	<0.001
household income	OS	0.065	0.008	7.922	<0.001
sex	migrating for offspring	0.038	0.009	4.401	<0.001

S.E., standard error; C.R., critical ratio (B/S.E.); CPE, community physical examinations; OS, outpatient services *, *p* < 0.05; **, *p* < 0.01; ***, *p* < 0.001.

**Table 6:** Path coefficients for the hypothetic model 2.

Path Constructs		Coefficient	S.E.	C.R.	*p*
Independent Variable	Dependent Variable				
migrating duration	migrating for offspring	−0.020	0.006	−3.566	<0.001
migrating duration	outer social support	0.066	0.019	3.424	<0.001
migrating for offspring	outer social support	−0.061	0.023	−2.630	0.009
outer social support	health insurance	0.111	0.033	3.382	<0.001
outer social support	local friends	0.384	0.099	3.867	<0.001
outer social support	OS	0.316	0.082	3.864	<0.001
migrating duration	CPE	−0.059	0.020	−2.982	0.003
migrating duration	OS	−0.035	0.008	−4.567	<0.001
migrating for offspring	CPE	−0.039	0.025	−1.595	0.036
migrating for offspring	OS	−0.028	0.013	−2.167	0.030
health status	CPE	0.019	0.007	2.723	0.006
health status	OS	0.001	0.008	0.095	0.924
household income	CPE	−0.038	0.008	−4.842	<0.001
household income	OS	0.065	0.008	7.922	<0.001
sex	migrating for offspring	0.038	0.009	4.401	<0.001

S.E., standard error; C.R., critical ratio; CPE, community physical examinations; OS, outpatient services *, *p* < 0.05; **, *p* < 0.01; ***, *p* < 0.001.

**Table 7:** Mediating effect test.

IV	M	DV	IV-DV	IV-M	(IV+M)-DV	
					IV	M
migrating for offspring	local friends	CPE	−0.029 **	−0.038 ***	−0.024 *	0.123 ***
migrating for offspring	local friends	OS	−0.030 **	−0.038 ***	−0.031 **	0.042 ***
migrating duration	health insurance	OS	−0.024 *	0.026 **	−0.024 *	−0.015 *
migrating duration	local friends	OS	−0.024 *	0.057 ***	−0.026 **	0.042 ***
migrating for offspring	sex	CPE	−0.029 **	0.035 ***	−0.029 **	0.010
migrating for offspring	sex	OS	0.030 **	0.035 ***	0.030 **	−0.004

IV= independent variable; M: mediator; DV: dependent variable; CPE, community physical examinations; OS, outpatient services *, *p* < 0.05; **, *p* < 0.01; ***, *p* < 0.001.

**Table 8:** Results of hypotheses testing.

Hypothesis	Results
H1: Migrating duration → the use of health services (negative)	Supported
H2: Migrating for offspring → the use of health services (positive)	Unsupported (negative)
H3: Outer social support → the use of health services (positive)	Supported
H4: Mediating effect of outer social support on the relationship between migrating duration and the use of health services	Supported
H5: Mediating effect of outer social support on the relationship between migrating for offspring and the use of health services	Supported

## Abbreviations

EPHS	essential public health services
CPE	community physical examinations
OS	outpatient services
SEM	structural equation modeling
C.R.	critical ratio
IV	independent variable
M	mediator
DV	dependent variable
